# Sex-Selective Effects on Behavior in a Mouse Model of Tuberous Sclerosis Complex

**DOI:** 10.1523/ENEURO.0379-19.2020

**Published:** 2020-04-29

**Authors:** Rachel Michelle Saré, Abigail Lemons, Christopher Figueroa, Alex Song, Merlin Levine, Carolyn Beebe Smith

**Affiliations:** Section on Neuroadaptation and Protein Metabolism, National Institute of Mental Health, National Institutes of Health, Department of Health and Human Services, Bethesda, MD 20814

**Keywords:** sex differences, tuberous sclerosis complex

## Abstract

Tuberous sclerosis complex (TSC) is an autosomal dominant genetic disorder that is caused by a mutation in either *TSC1* or *TSC2*. TSC affects multiple systems of the body, and patients with TSC display a range of neurologic and behavioral manifestations including seizures, intellectual disability, autism spectrum disorders, attention deficit hyperactivity disorder, anxiety, and mood disorders. Whereas behavioral phenotypes of many mouse models have been studied, the effects of sex have, for the most part, not been explored. We studied adult male and female *Tsc2* heterozygous and control mice to investigate the influence of sex and genotype on behavior. On a test of social preference, *Tsc2* heterozygous mice, regardless of sex, demonstrated lower preference for the stranger mouse than control mice. In the open field, *Tsc2* heterozygous males and control females habituated to the open field with decreasing anxiety-like behavior over time, whereas *Tsc2* heterozygous females did not show habituation to the open field environment. We did not find any statistically significant effects of genotype on open field activity, learning and memory or motor function. Our results highlight phenotype differences in *Tsc2* heterozygous mice, some of which are influenced by sex. A consideration of how sex influences the behavioral phenotypes of TSC is critical to develop a more complete understanding of the disorder and better target future pharmacological treatments.

## Significance Statement

We investigated the role of sex on behavioral phenotypes in a mouse model of tuberous sclerosis complex (TSC). Our findings reveal potentially important sex differences in habituation to an anxiety-provoking environment. These results provide a more complete understanding of the disorder and highlight the need to investigate sex-specific differences to better target treatment of the disorder.

## Introduction

Tuberous sclerosis complex (TSC) is an autosomal dominant neurogenetic disorder affecting approximately 1 in 6000 people. It is caused by a mutation in either *TSC1* or *TSC2* (European Chromosome 16 Tuberous Sclerosis Consortium, 1993; van Slegtenhorst et al., 1997). Multiple biological systems are affected in TSC, but the neurologic manifestations are often the most debilitating. *TSC1* and *TSC2* form a complex to inhibit the mammalian target of rapamycin (mTOR) pathway a regulatory node in cell growth and metabolism. The mTOR pathway is thought to play a key role in neuronal development and synaptic plasticity (Tavazoie et al., 2005; Jaworski and Sheng, 2006; de Vries and Howe, 2007), and consequently may be important in the unfolding of behavioral phenotypes.

Patients with TSC often experience seizures (80–90%), learning difficulties (50%), and autism spectrum disorders (50%; Hunt and Shepherd, 1993; Thiele, 2004). Moreover, mutations in *TSC2* tend to result in more severe neurologic phenotype (Au et al., 2007). Some studies of *Tsc2* heterozygous mice have reported deficits on learning and memory tasks (Ehninger et al., 2008; Auerbach et al., 2011; Tang et al., 2014), although other studies have not (Potter et al., 2013; Reith et al., 2013). *Tsc2* heterozygous mice have also displayed perseverative behavior (Potter et al., 2013) and social behavior abnormalities (Reith et al., 2013; Tang et al., 2014).

Sex may play a role in various phenotypes associated with TSC. Females are more likely to develop renal tumors than males (Hong et al., 2016). Lymphangioleiomyomatosis also occurs more commonly in female patients with TSC (Ryu et al., 2012). In addition, one epidemiological study indicated that male TSC patients were more prone to epilepsy and autism than female patients (Wataya-Kaneda et al., 2013); however, another study indicated that sex did not play a role in the central nervous system manifestations of TSC (Smalley, 1998). In most studies of mouse models of TSC, the effects of sex have not been investigated. Reported results are either from only male mice or from both sexes combined, but it is known that sex can have a profound role in both the morphology and function of the brain subsequently affecting behavioral output (Ngun et al., 2011).

In this study, we further investigated activity, anxiety-like behavior, social preference, learning and memory, and motor coordination in groups of male and female control and *Tsc2* heterozygous mice. Habituation to an anxiety-provoking environment (open-field) differed by genotype in a sex-dependent manner. We also found genotype differences in a test of social preference. We did not find genotype differences in open field activity, learning and memory, or motor function. Our results highlight important phenotypic differences, some of which are modulated by sex in *Tsc2* heterozygous mice and illustrate that sex differences should be considered in future behavioral studies.

## Materials and Methods

### Animals

*Tsc2* heterozygous mice (The Jackson Laboratory; B6; 129S4-*Tsc2^tm1Djk^*/J stock 004686) on a C57BL/6J background were maintained in house through mating of heterozygous and control breeder pairs. Animals were maintained in a climate-controlled central facility, on a standard 12/12 h light/dark cycle (lights on at 6 A.M.), with access to food and water *ad libitum*. was All procedures were approved by the National Institute of Mental Health animal care and use committee.

### Behavior testing

**Figure 1.**
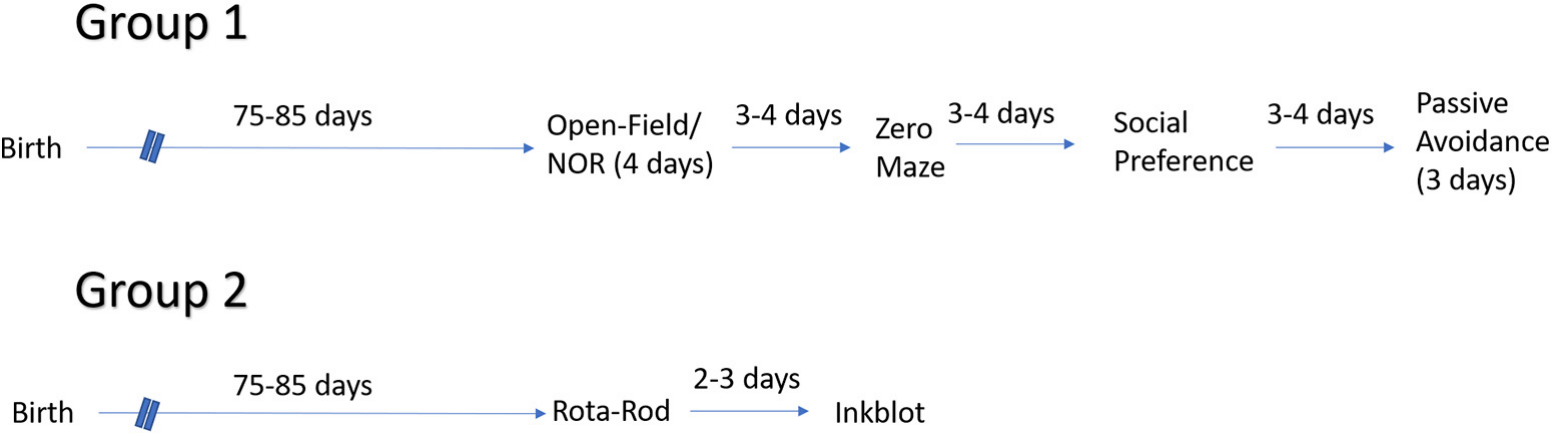
Timeline of testing. We started with two cohorts of mice.

Behavior testing began when the mice were between 75–85 d of age. All testing was done under normal lighting in the light phase. Testing was conducted in the following order: open field, novel object recognition (NOR), zero maze, social preference, and passive avoidance. Tests were spaced 3–4 d apart. We started with a cohort of 34 control males, 28 *Tsc2* heterozygous males, 40 control females, and 29 *Tsc2* heterozygous females. Every mouse underwent every test even if their data were not included. Data were not included because of prior exclusion criteria listed below, equipment malfunction, or testing performed outside of the time window presented. RotaRod and inkblot (spaced 2–3 d apart) analyses were performed in a separate group of animals starting with 24 control males, 25 *Tsc2* heterozygous males, 28 control females, and 30 *Tsc2* heterozygous females. An additional experiment to examine a methodological detail in the social preference assay was conducted on an additional 48 control males. The timeline of testing is shown (Fig. 1).

### Open field

To assess activity and anxiety-like behavior in response to a novel environment, mice were tested in an open field system (10.25-inch square clear Plexiglas chamber; Coulbourn Instruments) for 30 min. We analyzed data in 6-min epochs. Total horizontal distance traveled was used as a measure of activity and the ratio of distance traveled in the center (>6.25 cm from the wall) to total distance traveled was used as an inverse measure of anxiety-like behavior. Open field testing was performed between 9 A.M. and 1 P.M.

### NOR

To assess learning and memory, mice underwent NOR testing. Objects were colored plastic interlocking toy bricks or plastic bottles of sand. Objects were similarly sized, but different in shape and color. Testing was conducted in the open field arena, so the open field testing was the habituation phase of NOR (Day 1). At the same time of day, on Day 2, the animal was placed in the open field arena with two identical objects (plastic interlocking bricks or bottles of sand) on opposite ends of the arena. The animal explored the objects for 5 min. This training was repeated on Day 3. On Day 4, one of the objects was replaced by a novel object and the animal was allowed to explore for 5 min. All sessions were video recorded, and assessment of time spent sniffing each object was analyzed. If the animal spent <10-s sniffing in total, it was excluded as the error in the measurements to determine discrimination would be high. A discrimination index was calculated as the difference between time spent sniffing the novel object and time spent sniffing the familiar object divided by the total time spent sniffing.

### Zero maze

To assess anxiety-like behavior, the test mouse was placed on a zero maze (23.5-inch diameter ring with 2.75-inch wide beams; Med Associates) facing the open section of the maze and allowed to explore for 5 min. The amount of time spent in the open portion of the maze was determined. An animal was considered to be in a portion of the maze if both front paws were in that portion. If an animal fell off during the testing, it was eliminated from the analysis. Testing was performed between 12 and 5 P.M.

### Social preference

Social preference was assessed with a three-chambered apparatus (21.5 × 16-inch clear Plexiglas chamber equally divided into three compartments; Nadler et al., 2004). Testing was conducted in three parts (5 min each), with each phase immediately following the previous phase. (1) Habituation, the animal was habituated to the empty chamber. If an animal spent >3 min in one chamber, it was eliminated from the study for showing a side preference. We removed one control male, and three *Tsc2* heterozygous females that demonstrated a side preference during habituation. (2) Sociability, an age/sex matched control stranger mouse was placed inside a social enclosure (inverted wire cup) in one chamber and an empty social enclosure was placed in the opposite chamber. The test mouse was placed in the middle chamber and allowed to freely explore. (3) Preference for social novelty, a novel age/sex matched stranger mouse was placed in the previously empty social enclosure and the test mouse once again was allowed to freely explore. Sniffing (the animal’s nose directed toward the enclosure within 20 mm) was determined from video recordings with the TopScan software (Clever Systems). Testing was initiated between 12 and 5:30 P.M.

### Passive avoidance

Mice were tested on a passive avoidance system (14 × 7 × 12 inches; Coulbourn Instruments) with a 3-d protocol to assess learning and memory. Day 1, the animal was placed in the lighted chamber with the door to the dark chamber closed. After 30 s the door to the dark chamber was opened. Once the animal entered the dark chamber, the door closed automatically, and the test animal was returned to its home cage. Day 2, the animal was placed in the lighted chamber and after 30 s, the door to the dark chamber was opened. Once the animal entered the dark chamber, the door closed, and the animal received a footshock (0.3 mA, 1 s). The animal was kept in the dark chamber for 15 s and then moved to a holding cage for 120 s. The training session was then repeated, and the animal was returned to its home cage. Day 3, the animal was placed into the lighted chamber and after 30 s, the door to the dark chamber was opened. The latency to enter the dark chamber (maximum of 570 s) was recorded. Testing was performed at the same time of day across the testing sessions and occurred between 12 and 5 P.M.

### RotaRod

To assess motor function, mice were placed on an accelerating RotaRod (Columbus Instruments). The acceleration was set at 0.1 rpm/s. The time that the animal stayed on the RotaRod was measured and the average of two trials (1 h apart) was used for the analysis. Testing was initiated between 12 and 5 P.M.

### Inkblot

To assess gait, non-toxic ink was placed on the fore-(red) and hind-(black) paws of the mouse and the mouse was made to walk in a straight path along paper through a tunnel. The average length of the steps (measured from the heal of each hindpaw to the next) and the gait width was determined. Testing was performed between 1 and 5 P.M.

### Statistical analysis

The data are reported as mean ± SEM (standard error of the mean). Statistical analyses were performed using SPSS (IBM). NOR, zero maze, RotaRod, and passive avoidance data were analyzed by means of a two-way ANOVA with sex (male, female) and genotype (control, heterozygote) as between subjects’ variables. Data from open field, social preference, and inkblot were analyzed by means of a mixed-model repeated measures ANOVA with sex (male, female) and genotype (control, heterozygote) as between subjects’ variables and epoch (open field), chamber (social preference) and measure (inkblot) as within subjects’ variables. ANOVA results are reported in Table 1. When appropriate, *post hoc* differences were assessed by means of Bonferroni-corrected *t* tests. Effects with *p* ≤ 0.05 are considered statistically significant and are reported with a * symbol. Effects of 0.10 ≥ *p* > 0.05 are also reported and denoted with a ∼ symbol.

**Table 1:** ANOVA results

Behavior	Interaction	Main effect	*F*_(df,error)_ value	*p* value
Open field				
Total distance	Genotype × sex × epoch		*F*_(3,269)_ = 0.180	0.930
	Sex × epoch		*F*_(3,269)_ = 0.498	0.710
	Genotype × epoch		*F*_(3,269)_ = 0.244	0.890
	Genotype × sex		*F*_(1,78)_ = 2.288	0.134
		Genotype	*F*_(1,78)_ = 0.329	0.568
		Sex	*F*_(1,78)_ = 0.179	0.673
		Epoch	*F*_(3,269)_ = 151.442	<0.001*
Center/total distance ratio	Genotype × sex × epoch		*F*_(3,241)_ = 3.264	0.021*
	Sex × epoch		*F*_(3,241)_ = 0.180	0.914
	Genotype × epoch		*F*_(3,241)_ = 0.147	0.936
	Genotype × sex		*F*_(1,78)_ = 0.206	0.651
		Genotype	*F*_(1,78)_ = 0.594	0.443
		Sex	*F*_(1,78)_ = 10.500	0.002*
		Epoch	*F*_(3,241)_ = 2.749	0.042*
Zero maze	Genotype × sex		*F*_(1,64)_ = 0.151	0.699
		Genotype	*F*_(1,64)_ = 2.742	0.103
		Sex	*F*_(1,64)_ = 2.568	0.114
Social preference				
Sociability	Genotype × sex × chamber		*F*_(1,74)_ = 0.141	0.708
	Sex × chamber		*F*_(1,74)_ = 2.075	0.154
	Genotype × chamber		*F*_(1,74)_ = 3.916	0.052^∼^
	Genotype × sex		*F*_(1,74)_ = 2.733	0.103
		Genotype	*F*_(1,74)_ = 3.771	0.056^∼^
		Sex	*F*_(1,74)_ = 3.458	0.067^∼^
		Chamber	*F*_(1,74)_ = 83.014	<0.001*
Social novelty	Genotype × sex × chamber		*F*_(174)_ = 6.875	0.011*
	Sex × chamber		*F*_(1,74)_ = 4.785	0.032*
	Genotype × chamber		*F*_(1,74)_ = 0.118	0.732
	Genotype × sex		*F*_(1,74)_ = 11.392	0.01*
		Genotype	*F*_(1,74)_ = 4.076	0.047*
		Sex	*F*_(1,74)_ = 4.753	0.032*
		Chamber	*F*_(1,74)_ = 4.551	0.036*
NOR	Genotype × sex		*F*_(1,45)_ = 0.056	0.814
		Genotype	*F*_(1,45)_ = 0.213	0.646
		Sex	*F*_(1,45)_ = 0.083	0.774
Passive avoidance	Genotype × sex		*F*_(1,69)_ = 2.561	0.114
		Genotype	*F*_(1,69)_ = 0.224	0.637
		Sex	*F*_(1,69)_ = 4.558	0.036*
RotaRod	Genotype × sex		*F*_(1,76)_ = 0.388	0.535
		Genotype	*F*_(1,76)_ = 0.160	0.690
		Sex	*F*_(1,76)_ = 0.347	0.558
Inkblot				
	Genotype × sex × measure		*F*_(1,94)_ = 2.459	0.120
	Sex × measure		*F*_(1,94)_ = 2.381	0.126
	Genotype × measure		*F*_(1,94)_ = 0.468	0.496
	Genotype × sex		*F*_(1,94)_ = 1.417	0.237
		Genotype	*F*_(1,94)_ = 0.185	0.668
		Sex	*F*_(1,94)_ = 0.488	0.487
		Measure	*F*_(1,94)_ = 1842.215	<0.001*

## Results

### Open field activity

**Figure 2.**
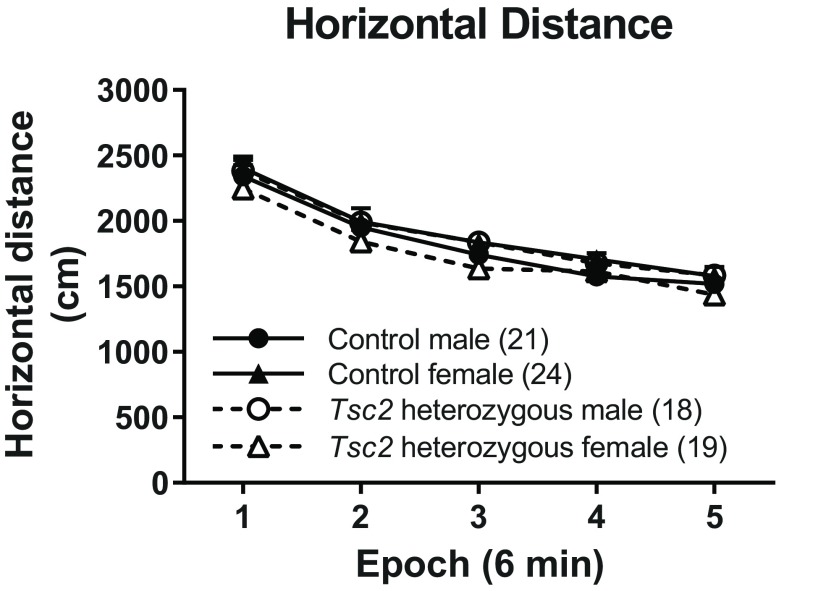
Activity levels. In distance traveled on the open field, we did not find any statistically significant main effects or interactions. Each point represents the mean ± SEM for the number of animals indicated in parentheses.

We tested mice in a novel open field environment. None of the interactions were statistically significant (Table 1). The main effect of epoch was statistically significant as would be expected as animals habituated to the novel environment over time (Fig. 2).

### Anxiety-like behavior

**Figure 3.**
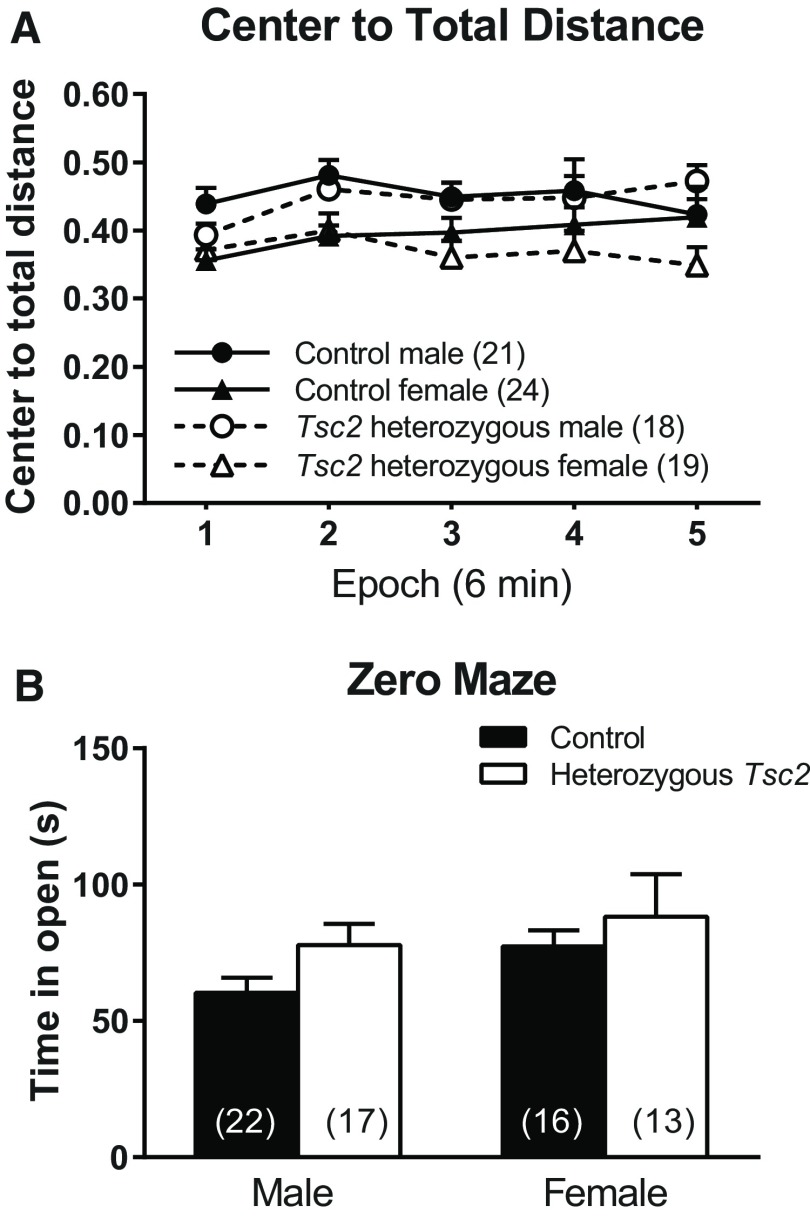
Anxiety-like behavior. ***A***, In the open field, we analyzed the ratio of distance traveled in the center of the open field to total distance traveled. The genotype × sex × epoch interaction was statistically significant. In control mice, males traveled more relative distance in the center than females in epochs 1 and 2 (*p* = 0.002 for each epoch). In *Tsc2* heterozygous mice, males traveled more relative distance in the center than females in epochs 2, 3, and 5 (*p* = 0.049, *p* = 0.015, and *p* = 0.008, respectively). Each point represents the mean ± SEM for the number of mice indicated in parentheses. ***B***, We also analyzed anxiety-like behavior with the zero maze by measuring the time in the open portion of the maze. There were no statistically significant interactions or main effects. Bars represent mean ± SEM for the number of animals indicated in parentheses.

To assay anxiety-like behavior, we measured the ratio of distance traveled in the center to total distance traveled in the open field (Fig. 3*A*) and behavior in the zero maze (Fig. 3*B*). In the open field, the sex × genotype × epoch interaction was statistically significant (*p* = 0.021; Table 1). *Post hoc* tests show that, in control mice, male mice traveled more relative distance in the center compared with female mice in epochs 1 and 2 (*p* = 0.002 for each epoch). In *Tsc2* heterozygous mice, male mice traveled more relative distance in the center compared with female mice in epochs 2, 3, and 5 (*p* = 0.049, *p* = 0.015, and *p* = 0.008, respectively; Fig. 3*A*). These data indicate that both *Tsc2* heterozygous males and control females showed habituation to the environment over time with decreasing anxiety-like behavior. *Tsc2* heterozygous females showed anxiety-like behavior at the beginning of the test and never habituated to the environment, whereas control males had less of an anxiety-like response to the novel environment and did not show habituation. In the zero maze, we found no statistically significant interactions or main effects of sex or genotype (Table 1; Fig. 3*B*).

### Social preference

**Figure 4.**
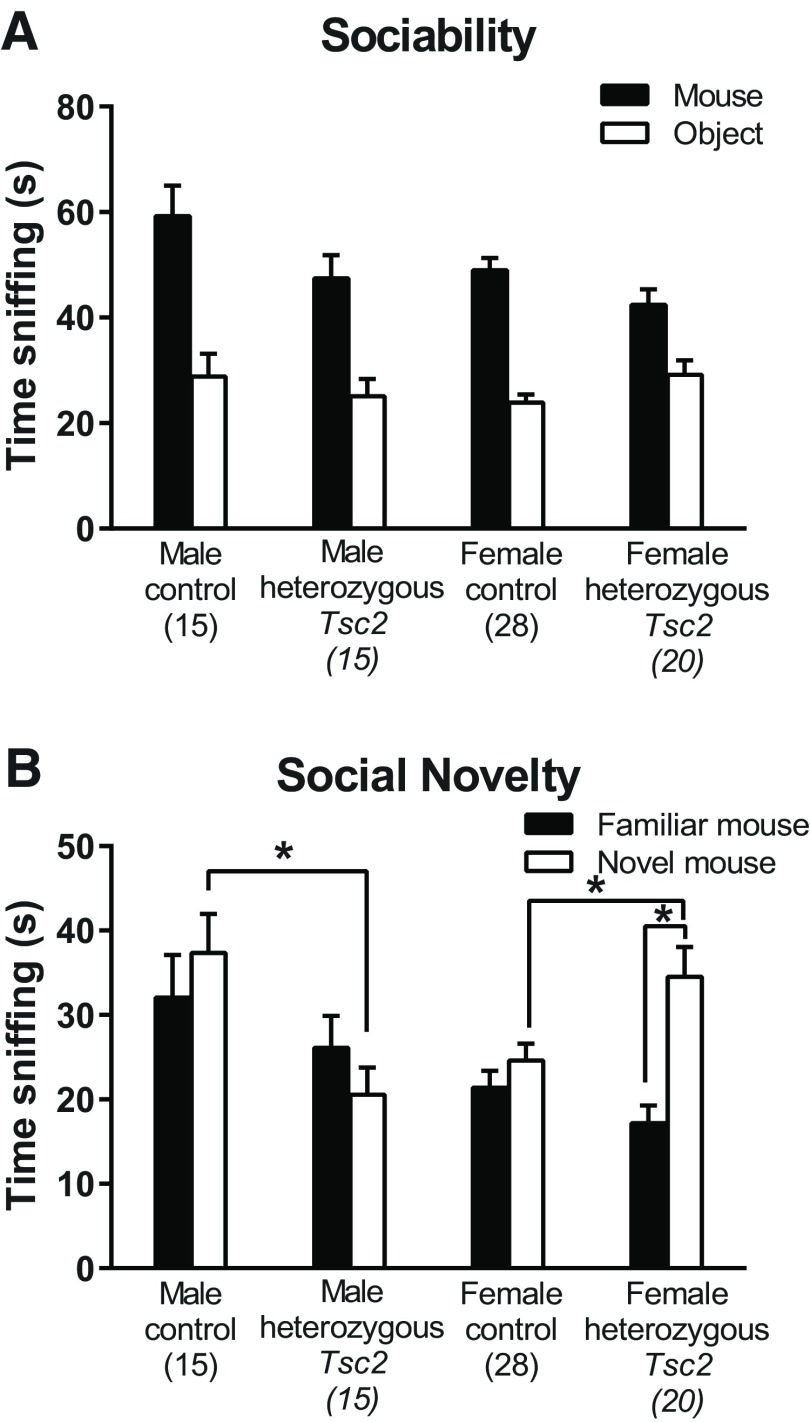
Social preference. ***A***, The chamber × genotype interaction approached statistical significance (*p* = 0.052). All mice, regardless of sex or genotype, sniffed the stranger mouse more than the object. *Post hoc* tests showed that control mice, regardless of sex, sniffed the social mouse more than *Tsc2* heterozygous mice did (*p* = 0.016). Bars represent mean ± SEM for the number of mice indicated in parentheses. ***B***, In the preference for social novelty test, the genotype × sex × chamber interaction was statistically significant (*p* = 0.011). Only *Tsc2* heterozygous females sniffed the novel mouse statistically significantly more than the familiar mouse (*p* < 0.001; paired *post hoc* tests). In males, *Tsc2* heterozygous mice spent less time sniffing the novel mouse compared with control mice (*p* = 0.002). In females, *Tsc2* heterozygous mice spent more time sniffing the novel mouse compared with control mice (*p* = 0.018). Other statistically significant effects (not indicated on the figure) are that control male mice spent more time sniffing both the familiar (*p* = 0.013) and novel (*p* = 0.006) mice compared with control female mice, and *Tsc2* heterozygous male mice spent less time sniffing the novel mouse than *Tsc2* heterozygous females (*p* = 0.005). Bars represent mean ± SEM for the number of mice indicated in parentheses. *p < 0.05.

We assayed social preference by means of a three-chambered social preference task. In the sociability phase in which the test mouse could choose to interact with either an object (the empty container) or a stranger mouse (Fig. 4*A*), the genotype × chamber interaction approached statistical significance (*p* = 0.052; Table 1) suggesting that, regardless of sex, control mice demonstrated a greater preference for the mouse over the object than the *Tsc2* heterozygous mice (*p* = 0.016; Fig. 4*A*).

In the preference for social novelty phase, the test mouse could interact with either the familiar mouse or a novel mouse (Fig. 4*B*); in this test, the genotype × sex × chamber interaction was statistically significant (*p* = 0.011; Table 1). *Post hoc* analyses revealed that only female *Tsc2* heterozygous mice spent more time sniffing the novel mouse than the familiar mouse (*p* < 0.001). Male *Tsc2* heterozygous mice showed a statistically significant reduced sniffing time for the novel mouse compared with controls (*p* = 0.002), whereas female *Tsc2* heterozygous mice showed a statistically significant increased time sniffing the novel mouse compared with controls (*p* = 0.018). Other statistically significant effects (not indicated on the figure; Fig. 4*B*) are that control male mice spent more time sniffing both the familiar (*p* = 0.013) and novel (*p* = 0.006) mice compared with control female mice, and *Tsc2* heterozygous male mice spent less time sniffing the novel mouse than *Tsc2* heterozygous females (*p* = 0.005).

### Learning and memory

**Figure 5.**
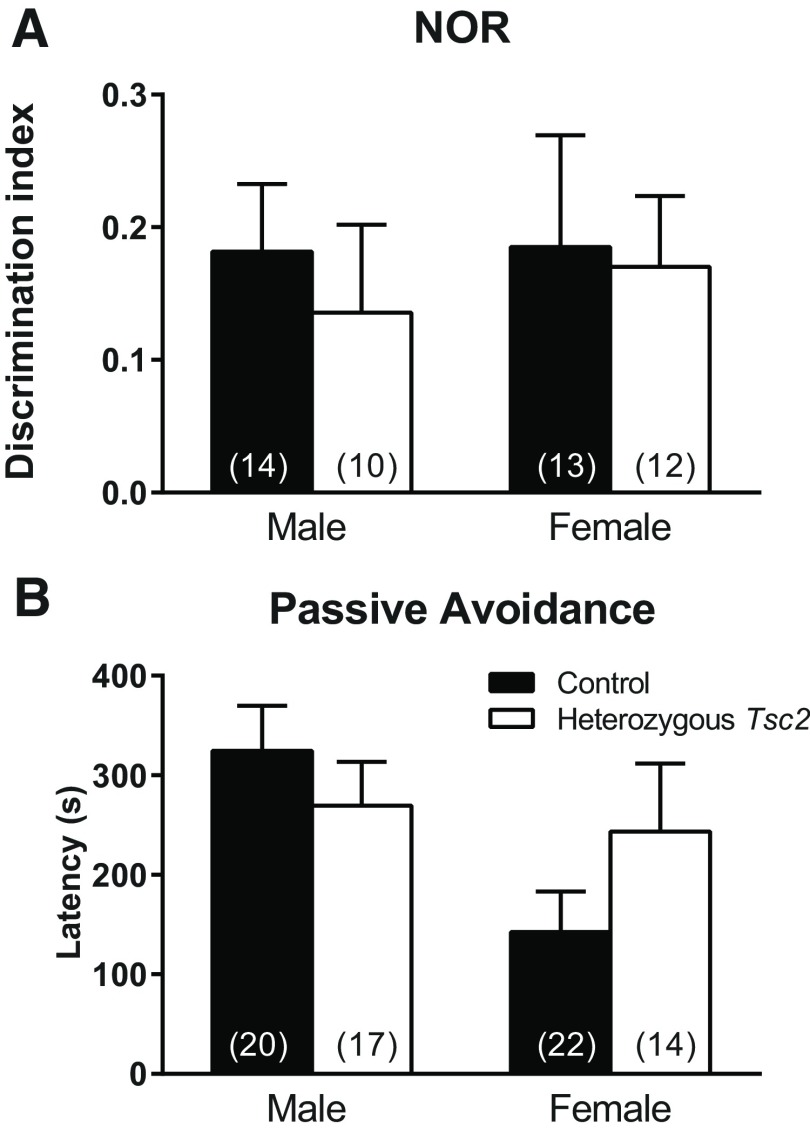
Learning and memory. ***A***, Results of NOR test do not show any statistically significant interaction or main effects. Bars represent mean ± SEM of the number of mice indicated in parentheses. ***B***, In the passive avoidance test, the main effect of sex was statistically significant (*p* = 0.036), but effects of genotype were not. Overall, regardless of genotype, females had a lower latency to enter the dark side than males. Bars represent mean ± SEM for the number of animals indicated in parentheses. The legend in ***B*** applies to both panels.

To assess learning and memory, we used NOR (Fig. 5*A*) and passive avoidance (Fig. 5*B*) tests. On the NOR, we did not find any statistically significant interactions or main effects (Table 1) likely due to the large variability in the measurements. On the passive avoidance task, we found a statistically significant (*p* = 0.036) main effect of sex, but no statistically significant effects with regard to genotype (Table 1) Overall, female mice, regardless of genotype, had a shorter latency than male mice (Fig. 5*B*).

### Motor function

**Figure 6.**
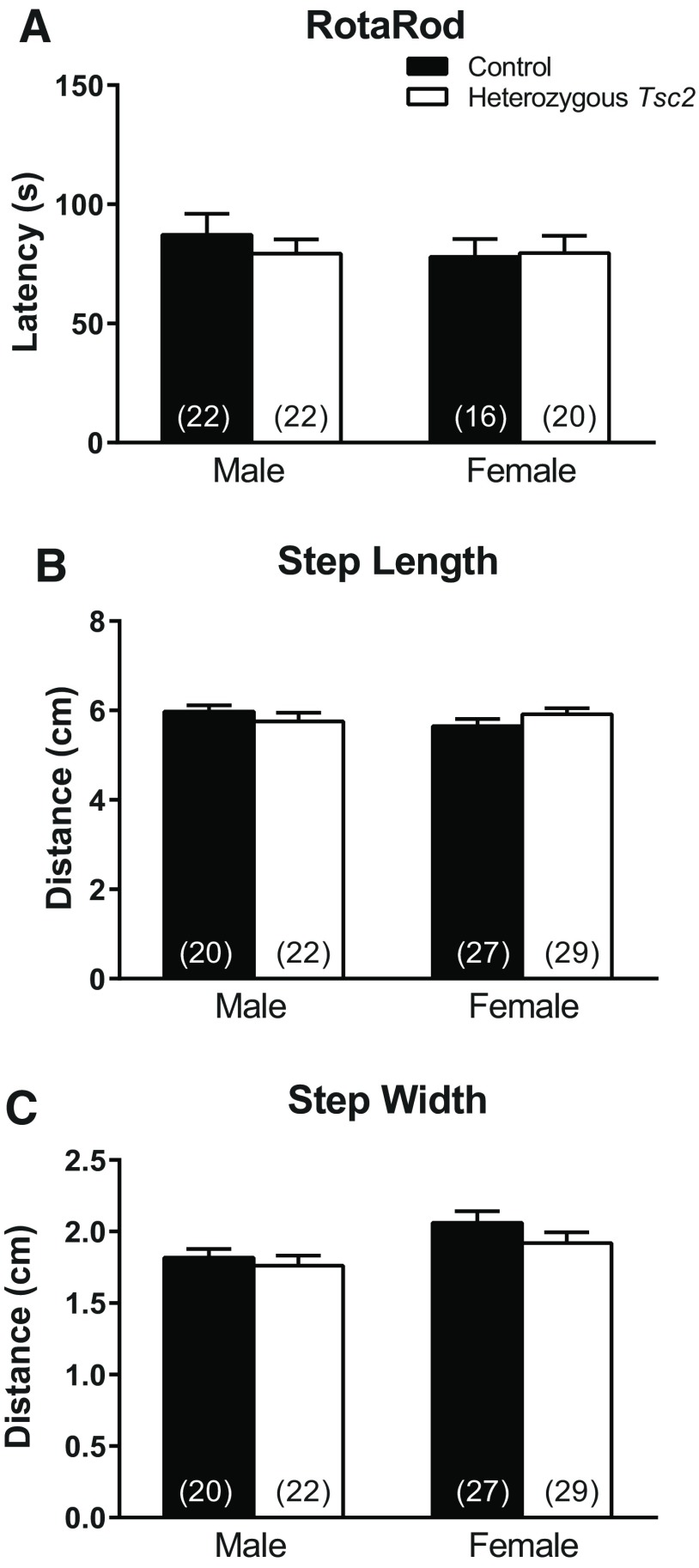
Motor function. ***A***, Average latencies to fall off an accelerating RotaRod after two trials do not show any statistically significant interactions or main effects. Bars represent mean ± SEM for the number of animals indicated in parentheses. ***B***, ***C***, Analysis of gait (step length and step width) show a statistically significant main effect of measure (*p* < 0.001) indicating that step width is less than step length. There were no statistically significant differences regarding sex or genotype. Bars represent mean ± SEM for the number of animals indicated in parentheses. The legend in ***A*** applies to all three panels.

On the zero-maze task, we noticed that *Tsc2* heterozygous mice tended to fall off more than control mice (11.4% of control males vs 21.4% of *Tsc2* heterozygous males and 10% of control females vs 20.7% of *Tsc2* heterozygous females). We sought to further investigate motor function/coordination with the RotaRod test (Fig. 6*A*) and the inkblot test (Fig. 6*B*,*C*) to measure gait width and step length. On the RotaRod test none of the interactions or main effects were statistically significant (Table 1; Fig. 6*A*). On the inkblot test, there were no statistically significant differences regarding sex or genotype (Table 1; Fig. 6*B*,*C*).

## Discussion

The present study is, to our knowledge, the first study of the *Tsc2* heterozygous mouse model of TSC to systematically examine sex differences in behavioral phenotype. In the open field, we saw differences in habituation to the novel environment with respect to anxiety-like behavior. *Tsc2* heterozygous males and control females habituated to the open field with decreasing anxiety-like behavior over time, whereas *Tsc2* heterozygous females did not show habituation to the open field environment. We also found genotype-specific differences in a test of social preference. We did not find any statistically significant effects of genotype on open field activity, learning and memory, or motor function. Our results highlight male/female differences in effects of a knock-out of a single allele of *Tsc2* on some behaviors.

**Figure 7.**
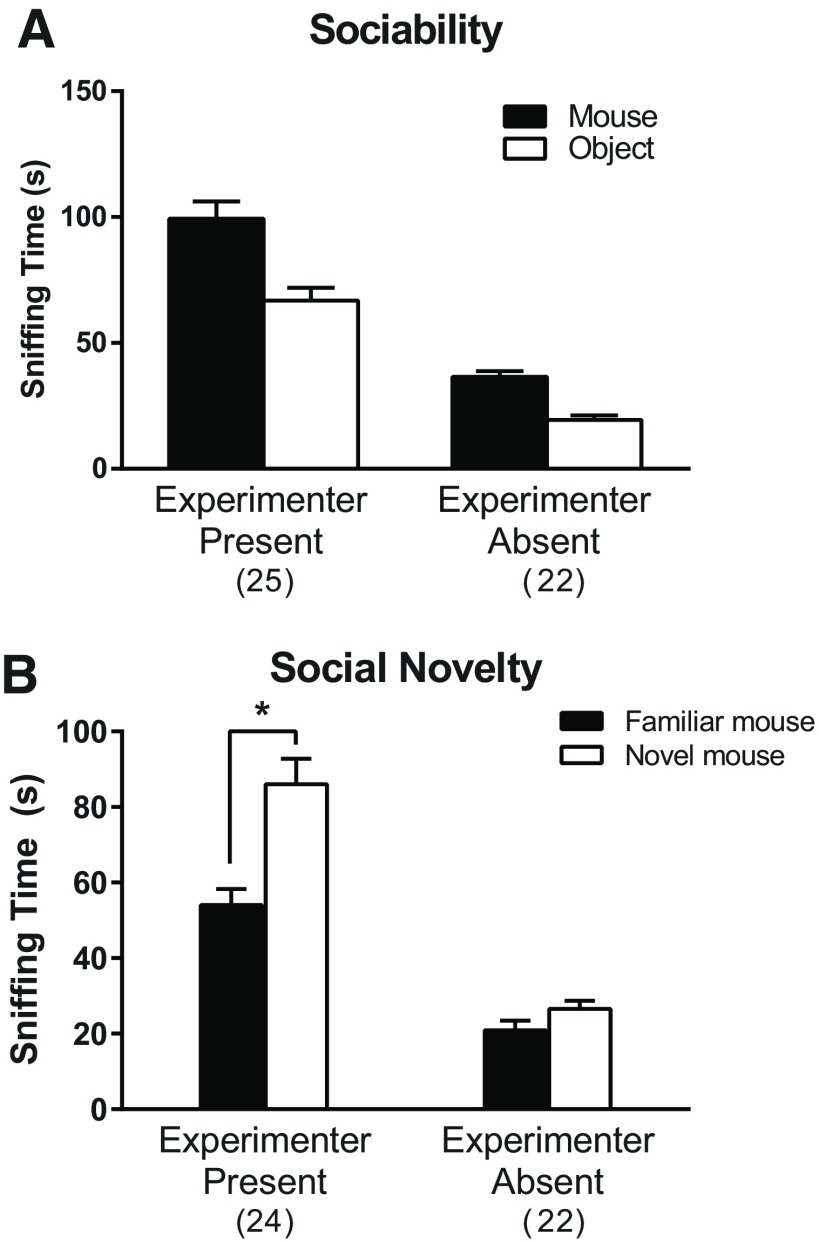
Social preference in control males in the presence or absence of the experimenter. We compared sociability and preference for social novelty whether or not the experimenter was present in the room (note: the male experimenter differed for each condition). ***A***, The experimenter presence × chamber interaction was not statistically significant, but both the main effect of experimenter presence (*F*_(1,45)_ = 243.1, *p* < 0.0001) and chamber (*F*_(1,45)_ = 18.72, *p* < 0.0001) were. ***B***, The experimenter presence × chamber interaction was statistically significant (*F*_(1,44)_ = 8.35, *p* = 0.006). *Post hoc t* tests showed that control males have a statistically significant preference for the novel mouse compared with the familiar mouse (*p* < 0.001) only if the experimenter is present. Bars represent mean ± SEM for the number of mice indicated in parentheses. We tested 26 mice with the experimenter present and 22 mice with the experimenter absent. Data from one mouse during the sociability phase was lost. During the social novelty phase with the experimenter present, we lost one mouse because the door closed on his tail following habituation and another mouse because someone entered the room during the test. *p < 0.001.

One of the limitations of our study is in the test of social behavior, specifically the response to social novelty. Neither male nor female control mice demonstrated a preference for the novel mouse. We have noted this behavior in prior studies in our lab. In the present study and in previous reports of a lack of social novelty preference, we note that the experimenter was not in the room during the test; mice were video recorded during the test, and results were analyzed later. In an ancillary experiment, we tested for an effect of the presence or absence of an experimenter present during the test. Results in control adult male C57BL/6J mice indicate that the preference for social novelty was demonstrated only when the experimenter was present (Fig. 7). It is interesting that in the original paper describing the three chambered task to measure preference for social novelty, the experimenter was present in the room (Nadler et al., 2004). This issue warrants further study.

Reports that did not account for sex did not find any anxiety-like behavior in *Tsc2* heterozygous mice (Ehninger et al., 2008, 2012; Potter et al., 2013; Tang et al., 2014), though *Tsc2* dominant negative mice (balanced mix of both sexes) did show a trend toward anxiety-like behavior as measured in the open-field (Ehninger and Silva, 2011). Our finding that female, but not male, *Tsc2* heterozygous mice did not habituate to the novel environment and maintained high anxiety levels throughout the test (did not adapt to the new environment) are of interest in view of the common occurrence of anxiety disorders in patients with TSC (25–60%; Lewis et al., 2004; de Vries et al., 2007; Muzykewicz et al., 2007; Pulsifer et al., 2007). We did not observe genotype differences in anxiety per se in either the open-field or zero-maze tests in mice of either sex.

One specific point that we aimed to address in the present study was whether there were learning and memory deficits in *Tsc2* heterozygous mice. It is generally believed that *Tsc2* heterozygous mice show learning and memory deficits, but not every study has reported an abnormality. Some studies have shown that with the Morris water maze (MWM) test, *Tsc1* or *Tsc2* heterozygous animals have reduced number of crossings during the probe trial (Goorden et al., 2007; Ehninger et al., 2008), but others have shown no genotype difference in *Tsc2* dominant negative mice on a C57BL/6 background (Chévere-Torres et al., 2012), *Tsc2* heterozygous mice on a C57BL/6J 129 mixed background (Reith et al., 2013), and in a *Tsc2* heterozygous rat model (Eker rats; Waltereit et al., 2011). In addition to MWM, learning and memory has been tested by means of the NOR test. Young *Tsc2* heterozygous mice (C57BL/6J background) sniffed the novel object less than controls 30 min after exposure suggesting a deficit in short-term memory (Tang et al., 2014), but *Tsc2* dominant negative mice (Chévere-Torres et al., 2012) nor *Tsc2* heterozygous rats (Eker; Waltereit et al., 2011) showed a deficit on NOR with a 24 h delay before testing (long-term memory). We used two tests of learning and memory: NOR (recognition memory) and passive avoidance (fear-based learning and memory), and we saw no genotype differences on either test. We note that variability was very large on both tests as is often the case for behavior tests. Finally, although we observed a high proportion of *Tsc2* heterozygous mice falling off the zero maze, we did not find any evidence of a deficit in motor performance. This is also consistent with previous reports. Baseline motor performance on a RotaRod test was not altered in *Tsc2* dominant negative mice (Chévere-Torres et al., 2012). Additionally, *Tsc1* and *Tsc2* heterozygous mice did not show any differences in rotarod across the trial period (Sato et al., 2012; Tsai et al., 2012; Reith et al., 2013).

Here, we demonstrate sex-specific differences in habituation to an anxiety-provoking environment. These results suggest that female *Tsc2* heterozygous mice do not habituate to a novel environment and maintain a high anxiety response. Our results highlight the importance of assessing males and females separately in behavioral testing. Our finding of some phenotypic differences in male and female *Tsc2* heterozygous mice may yield insight into treatments of the behavioral phenotype of the disorder.

## Synthesis

Reviewing Editor: Margaret McCarthy, Univ of Maryland School of Medicine Decisions are customarily a result of the Reviewing Editor and the peer reviewers coming together and discussing their recommendations until a consensus is reached. When revisions are invited, a fact-based synthesis statement explaining their decision and outlining what is needed to prepare a revision will be listed below. The following reviewer(s) agreed to reveal their identity: Matthew Paul, Deena Walker.

This manuscript investigates the sex-specific behavioral effects of TSC2 mutation with a focus on anxiety-related, social, and learning and memory behaviors. The authors found Tsc2 heterozygosity has sex-specific effects on memory function and social behaviors. The article is well written and the data are important for those interested in TSC2 in the nervous system in informing future work and experimental design.

Specific Comments

1. While it is interesting that there were sex differences in the time spent sniffing a novel animal in the social preference test, the control mice did not show a preference for the novel stimulus animal - e.g., more time spent sniffing the novel mouse compared to the familiar mouse. This raises concerns about how the test was conducted and makes the results difficult to interpret. What was the interval between phases? If it was too long, the familiar mouse may no longer be familiar. If it is short, then it is odd that the control mice are not behaving as if the familiar mouse is familiar. It is interesting that there are no baseline sex differences in the WT animals. Among other things, this might be due to the time of day that the behavior was conducted. Please include more information regarding experimental design and a discussion of the lack of baseline sex differences in the WT animals.

There are other issues with the description and discussion of the social preference test. The authors refer to the test as a Social Behavior test, but it is really a 3-chamber social preference test. The authors report their social behavior results as social preference. However, the data shown are time spent sniffing, which is different from preference. Details are missing - e.g., time interval between each phase, genotype of the stimulus mice, etc... (see also comment on methods details missing for all tests).

2. There was a significant interaction in the passive avoidance test, but post-hoc tests did not detect any differences between groups. This makes it difficult to make conclusions from these findings. In their post-hoc analysis of the passive avoidance test, did the authors test differences between male and female control animals? It appears as though female controls had a shorter latency than males and that this sex difference was eliminated in the heterozygous animals. If there truly were no differences in post-hoc tests, it may be premature to conclude that fear memory was more affected in Tsc2 females than males.

3. Many details are missing from the methods

a) Details of all testing arenas/mazes should be provided - dimensions, color, material, etc...

a) Were all animals tested at the same time of day? Time of day and lighting conditions that tests occurred should be provided. This is particularly important for the tests of anxiety-like behavior.

c) Details of the objects used in the Novel Object Recognition test should be provided.

d) How many outliers were there in each group/sex/condition?

e) It would help the reader if the authors state in the methods that sample sizes for each group are indicated in the figure legends.

f) Please indicate what time of day the behavior was conducted and include if the animals were tested during the light or dark phase. The methods mention a “standard 12:12 light cycle” but do not indicate when the lights came on.

4. Please include the which post hoc testing was used in the methods. While it was included in the results section it is unclear if there were any p-value corrections applied to the post-hoc t-tests.

5. What software package was used for the statistical analyses?

6. Please indicate how many animals were included in the behavioral testing in the beginning and how many animals were excluded using each criteria. From the methods, it appears that a number of criteria were used to exclude animals.

7. Figure 1 is not convincing of any differences other than epoch. Given that there was no effect of sex (P=0.952) or significant sex x genotype interaction (P=0.273), it seems odd to state in the results that the effect appeared due to lower levels in females. Either a more convincing graph should be shown or authors should remove this statement. Also, activity levels in the center of the open field test do not appear to drop very much in the open field. Was this test conducted in the dark phase of the light:dark cycle?

8. Figure 2A is also not convincing. There appears to be a high ratio of center activity. What were the dimensions of the center versus the surround? Looking at the data, I am skeptical that this is measuring anxiety - there should be less activity in the center.

9. The sample size for some groups in the learning and memory tests are quite low compared to other tests. Is this because of removal of outliers? This seems a bit extreme to remove so many animals from the analysis - female heterozygotes from 23 (in the open field test) to 13 (in the novel object recognition test).

Minor comments

1. Trends should not be reported with a symbol as this gives the impression of reaching significance. The reader is capable of simply reading the P-value in the table.

2. It would help the reader if the statistical test and details were included in the text, rather than simply stating the p-value.

3. There should be more discussion of Tsc2 mice in the introduction.

4. It would be helpful if the authors could expand on the difference in anxiety-related effects observed in their study and the literature in the discussion. Additionally, it would be helpful if the authors could put the results in a little more context with the literature. For example, why would TSC2 heterozygosity influence fear-based memory but not other types of memory? Do the authors have thoughts on this?

5. It might also be nice to include a schematic of the experimental design and the number of animals used in each behavioral test as a new figure or in the first figure. This would make it easier to understand the approach
